# Polar licit and illicit ingredients in dietary supplements: chemometric optimization of extraction and HILIC-MS/MS analysis

**DOI:** 10.1007/s00216-024-05173-4

**Published:** 2024-02-09

**Authors:** Matteo Baglietto, Barbara Benedetti, Marina Di Carro, Emanuele Magi

**Affiliations:** Department of Chemistry and Industrial Chemistry, University of Genoa, via Dodecaneso 31, 16146 Genoa, Italy

**Keywords:** Design of experiments, Salt-assisted liquid–liquid extraction, Doping substances, LC–MS/MS

## Abstract

Many dietary supplements claim the ability to enhance sports performance and to improve the fitness of the consumers. Occasionally, along with legal ingredients, illicit compounds may be added without being labelled, leading to unintended doping. Hence, the aim of this study was to develop an analytical method to determine a set of 12 polar (logD_pH=7_ from −2.0 to +0.3) compounds including diuretics, stimulants, β_2_-agonists, methylxanthines, and sweeteners. Hydrophilic interaction liquid chromatography was chosen as separation strategy, coupled with tandem mass spectrometry. The instrumental method was optimized using a two-step design of experiments (DoE). Firstly, a Plackett–Burman (PB) DoE was performed to identify the more influencing variables affecting peak areas and chromatographic resolution among temperature, water percentage in the mobile phase, and flow rate, as well as type and concentration of buffers. Secondly, a D-optimal DoE was set, considering only the most significant variables from the PB-DoE results, achieving a deeper understanding of the retention mechanism. Sample processing by salt-assisted liquid–liquid extraction was studied through DoE as well, and the whole method showed recoveries in the range 40–107% and procedural precision ≤11% for all analytes. Finally, it was applied to real samples, in which the four methylxanthines and two artificial sweeteners were detected and quantified in the range of 0.02–192 mg g^−1^. These values were compared to the quantities declared on the DS labels, when possible. Furthermore, a sequence of MS/MS scans allowed detection of a signal in one of the samples, structurally similar to the β_2_-agonist clenbuterol.

**Graphical Abstract:**

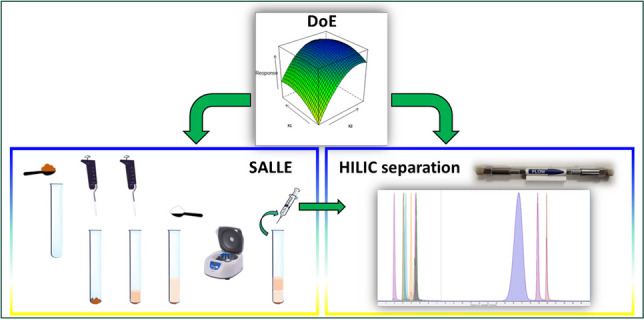

**Supplementary Information:**

The online version contains supplementary material available at 10.1007/s00216-024-05173-4.

## Introduction

Every year, the World Anti-Doping Agency (WADA) publishes and updates its own prohibited list, which includes the substances and methods prohibited for athletes both in and out of competition [1]. Furthermore, WADA has its World Anti-Doping Code (WADC) which harmonizes both anti-doping laboratory and bureaucratical procedures, and defines in detail whether a laboratory finding should turn into a sanction or not, when therapeutic exemptions are granted or unintended doping occurs [2].

Unintended doping is related to the undeclared presence of prohibited substances in allowed drugs [3], dietary supplements (DS) [4–8], and even food [5, 9]. This phenomenon could be either due to a cross contamination during the manufacturing process or to an illicit compounding by the producer, in order to sell “more effective” DS. Independently from the reasons of the presence of the prohibited substances, fair athletes would avoid suspicious products, and they are helped by some tools recently introduced. For example, lists of dietary supplements that claim or are revealed to contain substances prohibited by the WADA [10], or, on the contrary, databases with products certified as safe, after screening for over 1200 banned substances [11–13]. Nevertheless, if unintended doping is claimed by the athlete, counter-analyses of the suspected products (often DS) are necessary [5, 14].

The WADA’s list covers a wide range of compounds, with very different functionalities and physico-chemical properties [1]. Thus, careful optimization should be performed for a quantitative extraction of the analytes. Common strategies employed in the analysis of DS are liquid–liquid extractions or alkaline extractions, often followed by multiple laborious steps like clean-up and derivatization [8]. These approaches, usually applied to anti-doping analyses of urine, feed, or hair [15], also include solid-phase extractions and enzymatic hydrolyses [15–17]. Still, an ideal strategy should be greener, quicker, easier, and characterized by the same good process efficiencies [18]. Among these, ultrasound-assisted extraction [19, 20] and homogeneous liquid–liquid extraction (HLLE) are valid options. HLLE strategies involve the preparation of a single-phase solution, which allows for obtaining a theoretically infinite contact surface area between the sample and extraction solvent [21]. Then, an event induces a phase separation, and the fraction of interest could be even directly analyzed. Among HLLE, the use of switchable hydrophilicity solvents (SHS) [22] was successfully employed to extract some compounds including steroids and stimulants, mainly from human urine or foods [22, 23], even if not considering them directly as “doping substances.” Another HLLE approach is the acetonitrile extraction via salting-out effect (SOE), also known as salt-assisted liquid–liquid extraction (SALLE), that has been applied to various matrices and compounds [24], including caffeine, sweeteners, and diuretics, as well as β-blockers and their metabolites [25]. SOE is a phenomenon exploited also in the widely diffused QuEChERS (quick, easy, cheap, rugged, and safe) procedure, which was successfully applied to many analytes and matrices, including specific classes of doping substances in food and DS [26–28]. When dealing with different types of DS, including soft-gel-based supplements, complex matrix removal procedures could be necessary, employing strategies like dispersive liquid–liquid microextraction or enhanced matrix removal-lipid dispersive solid-phase extraction [29]. However, independently from the sample composition, matrix effects have to be checked along with the other method performances, especially if simpler and greener methods are developed.

Concerning instrumental analysis, historically, the most employed analytical strategies in doping controls relied on gas chromatography coupled with mass spectrometry (GC–MS) methods. Nowadays, except for some compounds determined by immunological procedures, most analytes are evaluated by GC or liquid chromatography (LC) coupled to tandem MS or high-resolution MS techniques [15]. Among the LC strategies, both reversed-phase LC (RPLC) [9, 30] and hydrophilic interaction liquid chromatography (HILIC) [31, 32] are applied in routine anti-doping controls. A recent study compared the two approaches for the analysis of sports drugs, evaluating the performances of different stationary phases for a wide range of compounds, and highlighting the complementarity of these separation techniques [33]. When analytes are characterized by high polarity and the presence of ionizable groups, the ideal separation strategy is HILIC chromatography, introduced by Alpert in 1990 [34]. Chromatographic separation, especially in HILIC mode, depends on many experimental variables, either related to column characteristics or operational conditions, which require to be controlled and optimized [35]. Thus, the multivariate approach through the design of experiments (DoE) is strongly recommended [35, 36]. DoE and other chemometric tools have already been used in HILIC studies, as recently reviewed by Taraji et al. [37]. In HILIC, the main parameters influencing the retention of a compound are type and percentage of organic solvent, salt content, pH, and column temperature, whose effects are strongly related to the specific stationary phase employed [37, 38].

In this study, a multivariate optimization was performed for the HILIC separation of 12 polar compounds and their SALLE extraction from DS. Substances prohibited by WADA’s list among the more frequently detected diuretics (hydrochlorothiazide and furosemide), stimulants (cocaine), and β_2_-agonists (salbutamol and clenbuterol) [39], as well as legal ingredients like artificial sweeteners and methylxanthines, were thus studied. The latter are not considered dopants for humans, but they are all included in the *Fédération Équestre International*’s prohibited list for horses [40]. Furthermore, not all competitions are under WADA’s control, and some of them are subject to more restrictive rules (e.g., NCAA [41]). Actually, caffeine was considered a prohibited substance by WADA between 1984 and 2004, when it was moved to the monitoring program [42]. Quantifying methylxanthines in DS is also important to avoid health risks. Indeed, a lot of caffeine-containing DS is consumed daily, sometimes ignoring the maximal daily intake of 400 mg (with a maximum of 200 mg per single dose) recommended by the authorities. So, it is worth quantifying its amount related to the suggested dose of DS, to monitor that this does not exceed the safety limit [6, 43].

To the best of the authors’ knowledge, this is the first application of SALLE to extract both typical ingredients and doping compounds from DS. The targeted analytes presented different physico-chemical properties, requiring an optimized multi-class method. After evaluation of the method performances, it was employed to process some DS bought online, allowing to detect and quantify some of the target analytes. Moreover, a sequence of MS/MS scans was performed to tentatively identify compounds structurally similar to the β_2_-agonist clenbuterol, which are less diffused and known but prohibited by WADA as well [1, 16, 17].

## Materials and methods

### Chemicals and reagents

All solvents and additives used were HPLC or LC–MS grade. Acetonitrile (ACN) and methanol (MeOH) were purchased from VWR (Fontenay-sous-Bois, France), while ultra-pure water was obtained in a lab using a Milli-Q Millipore (Watford, UK) system. Acetic acid (AA), formic acid (FA), and their ammonium salts (ANH_4_ and FNH_4_), evaluated as chromatographic additives, were supplied by VWR as well. Triethylamine (TEA) tested as a switchable hydrophilicity solvent was bought from Acros Organics (Geel, Belgium). Salts employed for SALLE were sodium chloride (purity ≥ 99%) from Sigma-Aldrich (St. Louis, MO, USA) and magnesium sulfate (99%) from Carlo Erba Reagenti (Rodano, MI, Italy). NaOH from Fluka Analytical (Saint Gallen, Switzerland) was employed along with AA as a pH modifier in both SHS-HLLE and SALLE.

**Fig. 1:**
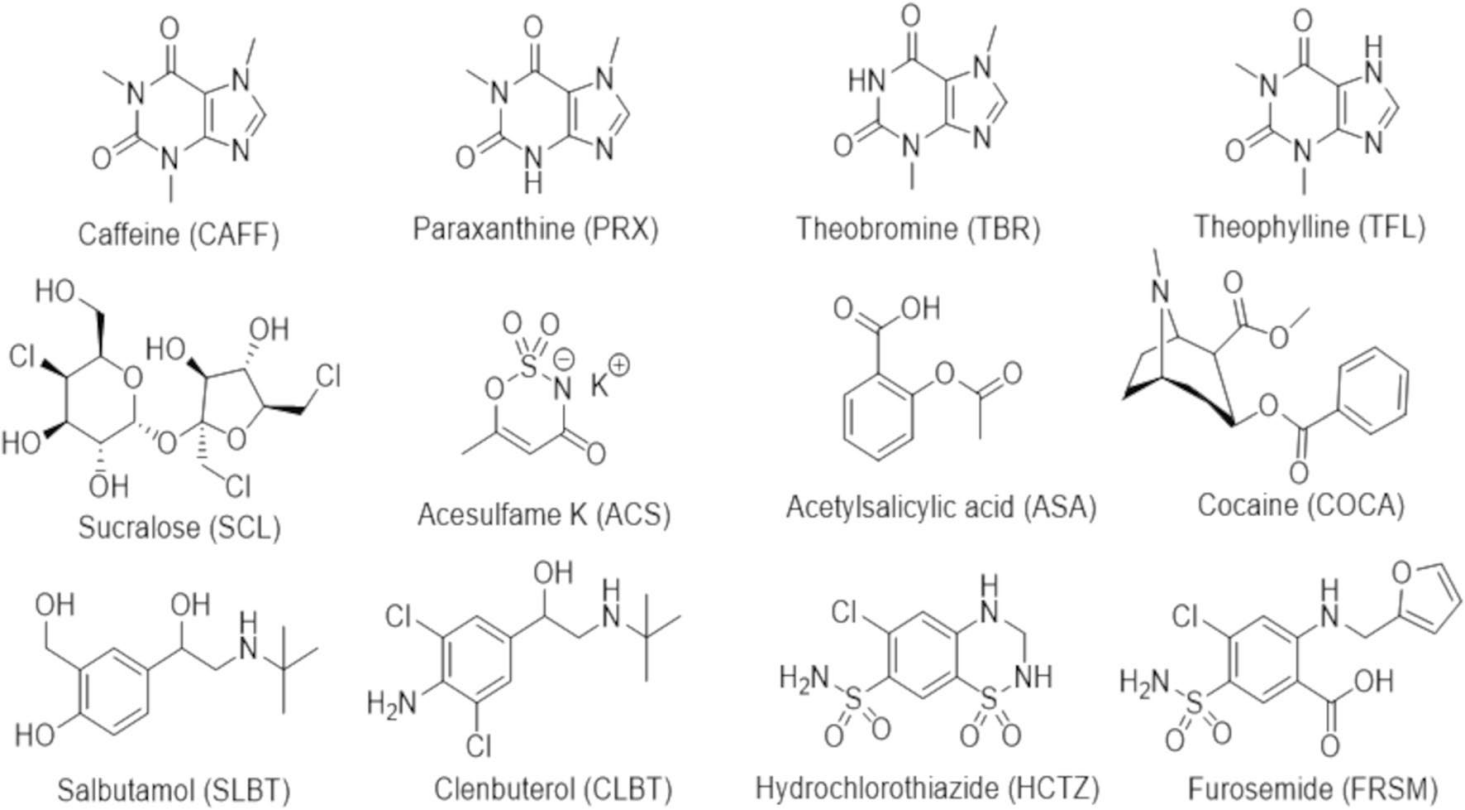
Molecular structures of the target analytes

Analytical standards (all above 98% of purity) were purchased from different suppliers: acesulfame (ACS), paraxanthine (PRX), theobromine (TBR), theophylline (TFL), sucralose (SCL), hydrochlorothiazide (HCTZ), furosemide (FRSM), cocaine (COCA), clenbuterol (CLBT), and acetylsalicylic acid (ASA) from Sigma-Aldrich; caffeine (CAFF) from Fluka Analytical; salbutamol (SLBT) from Alfa Aesar (Haverhill, MA, USA). The molecular structures of these target analytes are shown in Fig. 1. Stock solutions of single standards were prepared at a concentration ranging from 109 to 2720 mg L^−1^ in MeOH or MeOH:mQ water (1:1 v/v), and stored at − 18 °C (except for TBR and TFL at 4 °C and PRX at room temperature). A mixture of the standard solutions at 2 mg L^−1^ was obtained diluting single solutions and used to prepare daily working solutions by further dilutions.

### Design of experiments

The multivariate approach of DoE was employed to study both chromatographic separation and sample pre-treatment. As a first step, six chromatographic variables were considered: aqueous phase percentage in the eluent (A%: 0–10%), salt concentration (0.2–10 mM), acid percentage (0.01–0.1%) and buffer type (formate or acetate) within the mobile phase, column temperature (25–50 °C), and flow rate (0.1–0.3 mL min^−1^). Thus, a screening design was applied to investigate which of them was more relevant in influencing the separation, considering the retention of the selected analytes, their peak areas, and the chromatographic resolution as responses. Chromatographic resolution (R) between the peaks of substances “*a*” and “*b*” was calculated as follows:

1 $${R}_{ab}=2\cdot \frac{|R{T}_{a}-R{T}_{b}|}{\left({W}_{a}+{W}_{b}\right)}$$

where RT_a_ and RT_b_ are the retention times of the two peaks while *W*_a_ and *W*_b_ are their widths.

The selected screening design was a Plackett–Burman [44]: 8 experiments were performed, allowing the determination of the 7 coefficients of the linear model (one for each factor and the intercept). The coded values and experimental plan are summarized in Table S.1 (Supplementary information). Experiments were performed in random order to avoid any systematic drift affecting the results.

Results were elaborated by using the *Chemometric Agile Tool* (CAT) free software [45]. Through the computation of the models, the significance of the variables’ coefficients was assessed. The CAT software was also employed to implement the D-optimal algorithm, to choose the experiments to perform for the response-surface design considering the variables selected through the screening DoE. Based on the knowledge attained, only 4 variables were investigated at 3 levels (see Table S.2): aqueous phase percentage (A%: 0–10%) and salt concentration (0.2–10 mM) of the mobile phase, column temperature (20–45 °C), and flow rate (0.1–0.2 mL min^−1^). Among the set of all possible experiments, the D-optimal algorithm provides an indication of the best experiments to be performed [46, 47]. Thus, the 26 experiments reported in Table S.2 were selected and performed.

Once the instrumental method was established, the sample treatment strategy represented the next step to be optimized. In order to maximize the SALLE efficiency, five experimental variables were chosen based on previous knowledge [24, 25]: H_2_O:ACN ratio, amount of NaCl and MgSO_4_ employed to induce the SOE, pH, and centrifuge time. Thus, the complexity of the problem suggested once again the D-optimal DoE. The coded levels and respective real values of each variable are reported in Table S.3. Through the D-optimal algorithm, a set of 31 experiments was selected, and 5 of them were replicated as suggested by the “D-optimal by-Addition” algorithm [46], as reported in Table S.3. These experiments were performed by fixing the aqueous phase volume to 1.2 mL and by using NaOH or AA as pH modifiers.

### Instrumental analysis

A 1200 series HPLC coupled to a 6430 triple quadrupole mass spectrometer by Agilent Technologies (Santa Clara, CA, USA) was used to achieve chromatographic separation and analyte detection. The chromatograph was equipped with a binary pump, an online vacuum degasser, an automatic liquid sampler, and a thermostated column compartment. Separation was achieved on a YMC-Triart Diol-HILIC column (100 × 2.1 mm; 3 μm) by YMC Co. (Kyoto, Japan). Electrospray ionization (ESI) in a polarity switching mode was used as an ion source for the MS system. The following values were set for the ESI source: drying gas (N_2_) set at temperature and flow of 300 °C and 11 L min^−1^, respectively, nebulizer pressure 15 psi, and capillary voltage 4000 V. The multiple reaction monitoring (MRM) mode was employed during the chromatographic separation optimization and the targeted analyses. For most analytes, previously optimized parameters were used [48, 49]. The mass detection and fragmentation behavior of COCA, CLBT, HCTZ, and FRSM were studied by using the Mass Hunter Optimizer software (version B.04.01). For this purpose, 10 μL of single standard solutions, at a concentration of 500 μg L^−1^ in ACN:H_2_O (85:15, v/v), was repeatedly injected in the MS system in order to find the precursor and product ions, and to select the fragmentor voltage and collision energy (CE) which allow better sensitivity for each identified MRM transition. Data related to the MRM transitions for each analyte are summarized in Table S.4 (along with their physico-chemical properties): the most intense transition of each compound was used as the quantifier, while the ratio between the peak area of the qualifier (other characteristic MRM transitions) and the quantifier was used (when available) to confirm compound identity. The Agilent MassHunter workstation software (version B.04.01) was employed for data acquisition and qualitative and quantitative analysis.

Furthermore, aiming to screen CLBT analogues in suspicious DS, a precursor ion scan (PcIS) and a product ion scan (PdIS) were performed. To increase the sensitivity, a further MRM analysis was set, monitoring the most interesting transitions. The instrumental conditions of these scans are summarized in Table S.5.

After the chemometric optimization of the starting conditions, the following elution gradient was used for the chromatographic separation: eluent A was ultra-pure water with 0.01% FA and 0.2 mM FNH_4_, while eluent B was ACN:H_2_O 95:5 with the same amount of modifiers while column temperature was set at 25 °C; the combined eluent and flow gradient reported in Table 1 was employed to keep a good resolution within a reasonable run time of 25 min, including the restoration of the initial conditions. An experimental chromatogram of a neat standard is reported in Figure S.1.

**Table 1:** Gradient of the optimized method and retention times of the 12 target analytes

%A	%B	*t* (min)	Flow (mL min^−1^)	Retention times (min)			
0	100	0	0.1	Analyte	RT	Analyte	RT
0	100	5.5	0.1	ACS	1.99	SCL	4.52
0	100	7	0.3	FRSM	3.08	TBR	4.56
0	100	10	0.3	ASA	3.15	PRX	4.62
36.8	63.2	12	0.23	HCTZ	3.36	COCA	17.8
0	100	14	0.23	CAFF	4.00	CLBT	19.0
0	100	15	0.1	TFL	4.39	SLBT	20.0

### Dietary supplement samples

Eight different dietary supplements were bought on the Internet from specialized suppliers. None of them reported prohibited compounds on their label; among the declared ingredients were sweeteners, caffeine, and other methylxanthines.

Five of them were in a “capsuled” form (namely C1, C2, C3, C4, C5), and are supposed to be administered swallowing the capsules. The remaining three DS were in a powdered form (referred to as P1, P2, and P3), which are supposed to be solubilized in water and drunk. The first three supplements were used to prepare an equally weighted sample pool, removing the external coating and mixing the internal powders. This pool was stored at room temperature, as well as the samples, and it was employed to evaluate method performances, processing both spiked and non-spiked aliquots.

### Sample processing strategy

An aliquot of about 120 mg of sample was weighted in a falcon and added with 1.2 mL of milli-Q water with no pH modifier pre-warmed at 40 °C. Then, this was vortexed (using a VM3 vortex from CAT—Staufen, Germany) for 1 min at 2000 rpm, prior to adding 1.2 mL of ACN and further vortexing for 1 min at 2000 rpm. After that, the falcons were centrifugated for 6 min at 3500 rpm (using an ALC centrifugette 4206 from Aiken Corporation – Aiken, SC, USA), and the surnatant was transferred to another falcon containing 264 mg of NaCl and 1008 mg of MgSO_4_ (to have an overall concentration of 110 and 420 mg mL^−1^, respectively). This led to phase separation due to the SOE. After a further centrifugation for 9 min at 3500 rpm, the surnatant (organic phase) was withdrawn, filtered on 0.22 μm hydrophilic-PolyTetraFluoroEthylene filters (Thermo Scientific—San Jose, CA, USA), and then stored frozen at − 18 °C until analysis. Proper dilutions were performed before LC–MS/MS analysis, depending on the samples.

### Evaluation of method performances

Instrumental performances of the optimized method were evaluated in terms of linear dynamic range, in-matrix limits of detection and quantification, specificity, and intra-day and inter-day precision, while the sample processing protocol was evaluated in terms of recovery, repeatability, and matrix effect.

Calibration curves were constructed by analyzing blank matrices diluted in H_2_O:ACN, 5:95 (v/v) and spiked with analytes’ concentrations ranging from 0.05 to 50 μg L^−1^, to investigate the linearity range. In matrix limits of detection (LOD) and quantification (LOQ) were assessed in two ways considering the dilution of the samples. First, using the following proportion:

2 $$n \cdot {S}_{BK} :{C}_{LOD/LOQ}= {S}_{MS} : {C}_{MS}$$

where ***n*** is 3 for LOD and 10 for LOQ, ***S***_***BK***_ and ***S***_***MS***_ are the average signals (peak areas) of the matrix blanks and of the matrices spiked, respectively, while ***C***_***LOD/LOQ***_ and ***C***_***MS***_ are the corresponding concentrations. Otherwise, they were also evaluated using the equation:

3 $${C}_{LOD/LOQ}= n\frac{s}{b}$$

where ***s*** represents the standard deviation of the matrix blanks’ areas and ***b*** is the angular coefficient of the calibration curve [50]. As strongly recommended, the most conservative values were considered [50].

Specificity was assessed by verifying retention times of the MRM signals and, when possible, by calculating the ratios between the qualifier and quantifier transitions of each compound, monitoring that they maintain a deviation within a ± 30% from that of a reference standard as recommended by some guidelines [51].

Intra-day and inter-day instrumental precision were assessed at two concentration levels, 0.5 and 20 μg L^−1^ (except for COCA, at 0.05 and 2 μg L^−1^), chosen to provide a low and high value with sufficient sensitivity for most of the analytes as well as remaining within the linearity range. The relative standard deviation (RSD%) of peak areas of six replicates per level and during three non-consecutive days was calculated.

Sample treatment repeatability (procedural precision) was assessed as the RSD% of the analytes’ recoveries. They were calculated by processing six replicates of a spiked matrix (a real sample which showed no signals of any of the compounds studied [19]) and analyzing a properly diluted aliquot. Along with them, a “non-spiked” aliquot was processed, to evaluate the possible contribution of analytes present within the matrix. Three types of samples were thus analyzed: the non-spiked diluted aliquot (NS), the same aliquot, spiked after the processing with an amount corresponding to the quantitative recovery of the analytes (SA), and the aliquots spiked before processing (SB).

In this way, the analytes’ recovery can be evaluated as follows [52]:

4 $$R \left(\%\right)=\frac{{S}_{SB}-{S}_{NS}}{{S}_{SA}-{S}_{NS}} \cdot 100\%$$

where *S*_SB_, *S*_SA_, and *S*_NS_ are the analytical signals (peak areas) of the three types of samples analyzed.

After that, the matrix effect (ME) was assessed through the comparison (ratio) of the slopes of the calibration curves constructed by analyzing two series of standards, in-matrix and in pure solvent [53].

Finally, the greenness of the final methodology was assessed by using the Analytical Greenness (AGREE) software [54], and compared with those of other studies on DS with at least one analyte in common with this work.

## Results and discussion

### Development of HILIC method

Chromatographic separation involves a great number of experimental variables, whose effects depend on the selected compounds that have to be separated and the instrumental approach [35]. These effects are often interrelated and there is no “rule of thumb” universally valid. Thus, the investigation of these variables once at a time would not enable a deep understanding of the retention mechanism, besides the difficulties in reaching the true “optimum,” which can be more easily and rationally found with a multivariate approach [47].

Like the other chromatographic techniques, HILIC separation depends on several variables [37, 38, 55], whose effects are analyte-specific and column-depending [37, 38]. In this study, the separation of the selected analytes was evaluated on a diolic column, which contains a stationary phase with high hydrogen-bonding properties and low electrostatic-bonding properties, since it does not contain ionizable groups other than unreacted residual silanols [56].

First, a Plackett–Burman screening design was set, to highlight the main influencing variables (see “Plackett–Burman design—screening of the experimental variables”). Second, a D-optimal response-surface design was applied to deeply understand the effect of the selected variables and their possible influence (section “D-optimal design—understanding the mechanism”). During these steps, only isocratic elutions were considered, aiming to keep both experiment performance and interpretation of results as simple as possible. Finally, these optimized starting conditions were employed, exploiting the information previously achieved, when developing the final gradient separation.

#### Plackett–Burman design—screening of the experimental variables

Many responses have been modelled to minimize the time of analysis (12 retention times, RT), maximizing sensitivity (12 peak areas), and chromatographic resolution elaborating data obtained performing the PB-DoE. By performing a principal component analysis (PCA) on the obtained data matrix, more than 80% of the variance was explained by the first 3 PCs. The correlation of the variables changes with the analytes’ retention behavior being highlighted, due to the high fraction of variance explained. By labelling the experiments projected in the PC space depending on the coded levels of an experimental variable, they can be visually separated, when the selected variable resulted significant (see Figure S.2).

Useful information could be extrapolated by the computed models. Table 2 shows the coefficient significance and sign of the 12 retention time models, while Table S.6 reports the information on the peak areas models.

**Table 2:** Significance and sign of the coefficients of each variable in determining the retention time of each analyte. One, two, or three asterisks for *p* value < 0.05, < 0.01, < 0.001, respectively; *NS* not significant

Analyte	*A*%	[Salt]	%Acid	*T*	Flow	Buffer
ACS	** < 0	*** > 0	*** < 0	*** > 0	*** < 0	*** < 0
HCTZ	*** < 0	** > 0	NS	NS	*** < 0	NS
FRSM	*** < 0	*** > 0	NS	* > 0	*** < 0	*** > 0
CAFF	*** < 0	*** < 0	NS	NS	*** < 0	NS
TBR	*** < 0	NS	NS	** < 0	*** < 0	NS
PRX	*** < 0	NS	NS	NS	*** < 0	NS
TFL	*** < 0	NS	NS	NS	*** < 0	NS
SCL	*** < 0	*** > 0	* < 0	*** < 0	*** < 0	*** > 0
ASA	*** < 0	*** > 0	NS	*** > 0	*** < 0	*** > 0
COCA	*** < 0	*** < 0	** < 0	*** < 0	*** < 0	*** < 0
CLBT	*** < 0	*** < 0	NS	*** < 0	*** < 0	* < 0
SLBT	*** < 0	** < 0	NS	** < 0	*** < 0	NS

Among the studied variables, acid% was very often not significant in determining the retention of the analytes, even though it provided better sensitivity for some of them (Table S.6); buffer type was not significant in half of the retention models, while, when significant, it presented different effects. Moreover, acetate buffer allowed higher sensitivity for most of the negative-ionizing compounds (which are the less sensitive), as deduced by Table S.6. The remaining variables presented heterogeneous effects in determining the retention (and thus the resolution) of the analytes. Salt concentration presented the most divergent effects on the selected analytes. On the other hand, most of the significant coefficients of temperature were negative, while flow rate presented a strong and negative effect on retentions.

Regarding sensitivity, a well-known aspect of ESI was confirmed: both A% and flow showed a negative effect on peak area [57]. When significant, and with the exception of ACS and SCL, temperature and salt concentration presented a negative effect as well [58]. Indeed, since SCL is monitored as the acetate or formate adduct, increasing the salt concentration within the mobile phase increases the probability of producing the adduct within the ion source. Nevertheless, salt concentration was crucial in determining the resolution, so it was kept within the considered range in the following design.

All these considerations are valid only within the experimental domain, and the linear mathematical models required validation through the performance of the center point experiment. Only a few of these models were validated, highlighting the presence of significant interactions and quadratic terms, and thus suggesting a deeper investigation of the considered variables.

#### D-optimal design—understanding the mechanism

It is worth noticing that the conditions of experiment number 8 of the PB-DoE already allowed a satisfactory separation. Still, a further multivariate study was performed to achieve a better knowledge of the retention mechanisms, also evaluating the quadratic and interaction terms. In particular, the knowledge obtained by the PB was useful to narrow the experimental domain by removing the non-significant variables and by adjusting the ranges of the others. In fact, both acid% and buffer type were fixed (to 0.1% and acetate buffer, respectively, due to the sensitivity considerations previously done), also because keeping the same buffer within the chromatographic system accelerates the equilibration times, often very tedious in HILIC [58]. The remaining 4 variables were kept, but some ranges were adjusted: temperature range was lowered to 20–45 °C (also to extend the lifetime of the column), while flow rate was limited to 0.1–0.2 mL min^−1^, since its strong and negative effect could have overwhelmed the others (like that of A%). Indeed, A% and salt concentration ranges were kept as before.

**Fig. 2:**
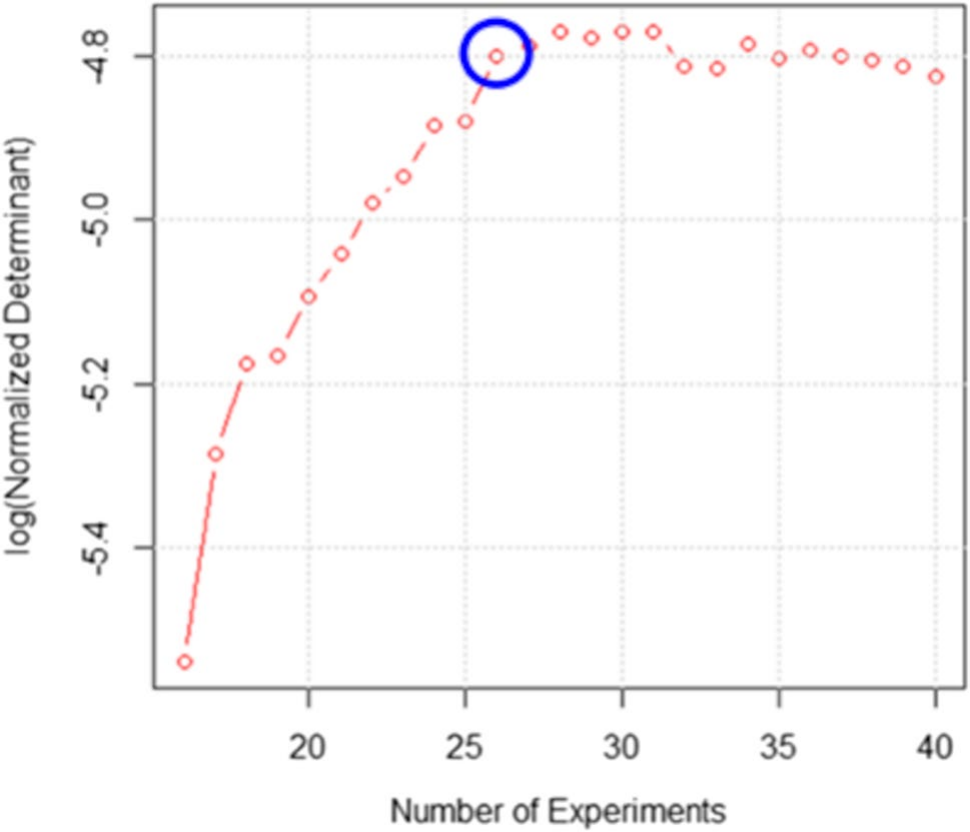
Trend of the log(normalizedDeterminant) based on the number of experiments in the subsets considered. Highlighted with a blue circle, the point chosen (the subset which included 26 experiments)

The D-optimal algorithm was used to select the best subset of experiments among the 3^4^ = 81 possible combinations of the experimental variables [46]. By applying this algorithm, it is possible to choose which terms are necessary in the response surface and the consequent best compromise between the information needed and experimental effort. Figure 2 represents the logarithmic normalized determinant of the information matrix of the experiments’ subset versus the number of experiments of each subset [46]. The log(normD) should be maximized, and in this case, 26 experiments (Table S.2) represented a good compromise, since this value is placed at the beginning of a *plateau* in the log(normD) graph. Moreover, the variance inflation factors, an indication of the design “goodness” in terms of minimal covariance among the coefficients to be computed, were satisfactory (< 2) for all the model coefficients.

**Fig. 3:**
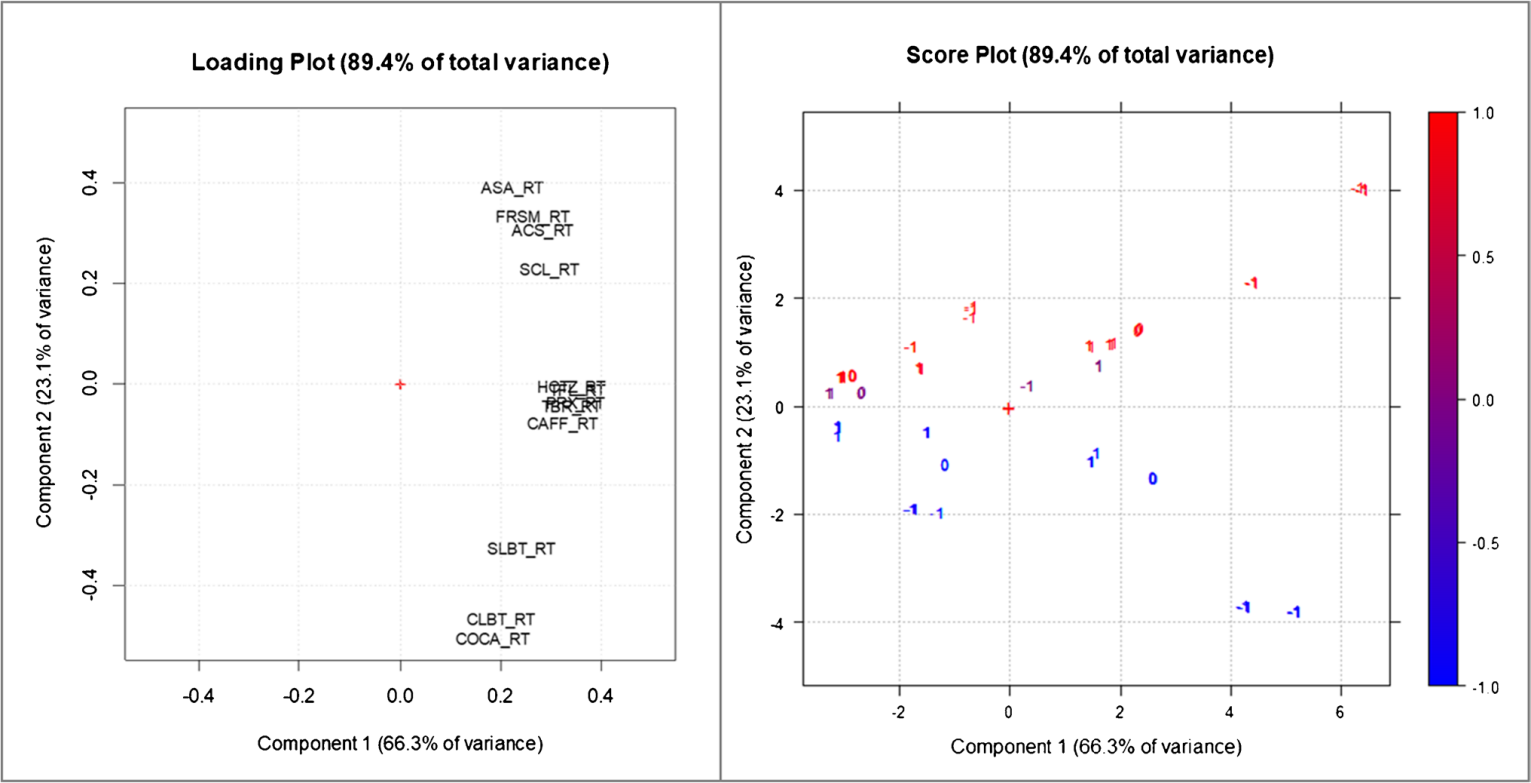
Loading and score plot related to the PCA performed on retention time data. The − 1, 0, and + 1 labels on the score plot correspond to the coded flow levels, while the color scale (legend on the right side) refers to the salt concentration coded levels

Due to the complexity of the dataset, results on RT and on resolutions were evaluated through two separate PCAs. Results of the RT-PCA are shown in Fig. 3, which reports both the loading and score plots. The first two PCs explained a high variance of the original data (89.4%): while the loadings on PC1 were always positive and characterized by a restricted distribution, the analytes were widely distributed along PC2. By looking at the score plot, in which the experiments are represented, PC1 correlated to the flow rate (as deduced from the numeric labels in the score plot). Instead, PC2 values were highly related to the salt concentration, indicated by the color scale in Fig. 3. Therefore, the PCA outcomes clearly showed which variables were crucial for the separation, as already deduced in the screening design. In particular, compounds can be visually divided into 3 groups, based on the effect that salt concentration had on their RT: at higher salt concentration, ASA, FRSM, ACS, and SCL were more retained, while SLBT, CLBT, and COCA eluted more quickly and the remaining compounds were less affected.

**Fig. 4:**
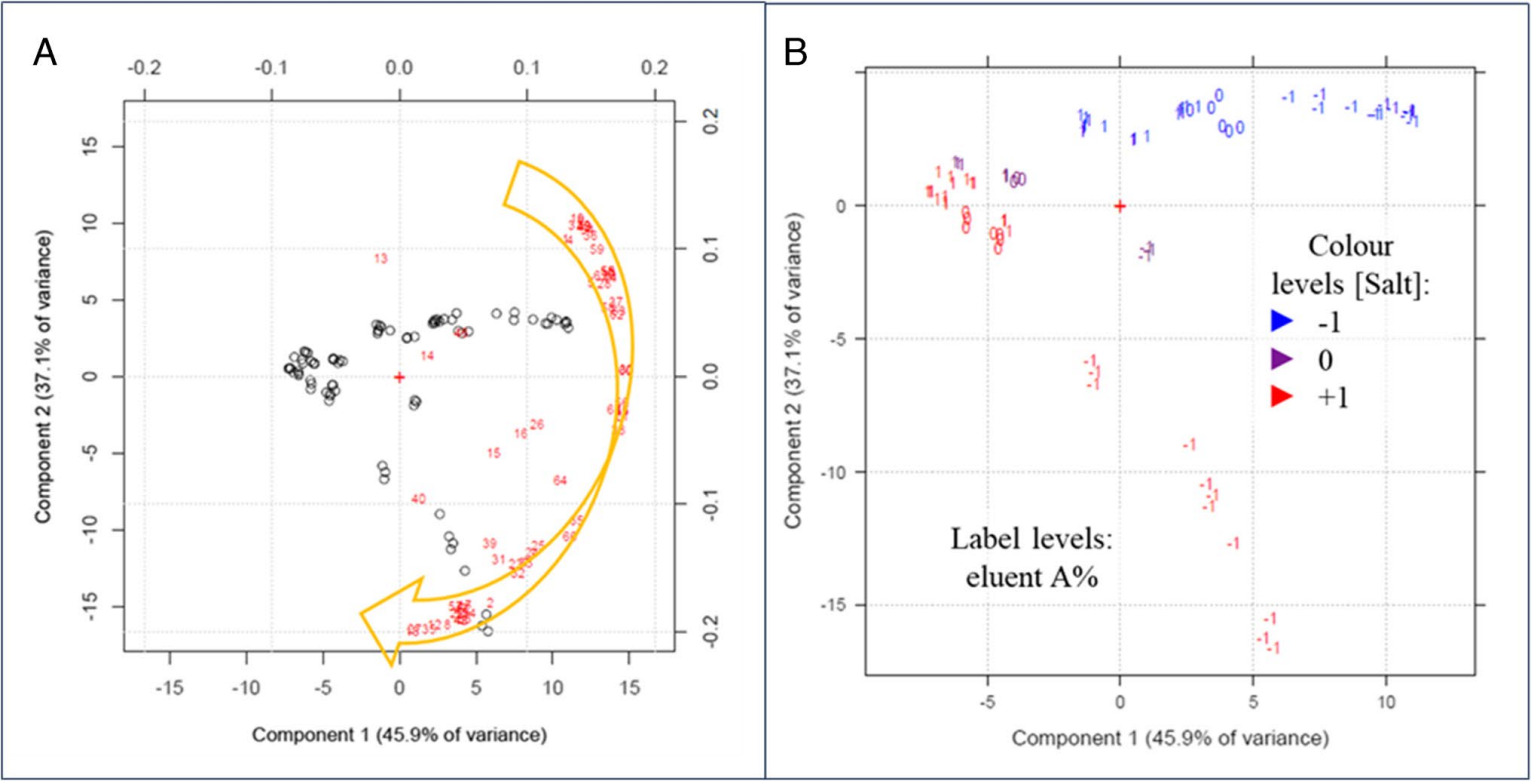
Biplot (**A**) and score plot (**B**) related to the PCA performed on the resolution data. Moving along the golden arrow, the salt concentration coefficient of the computed models gradually increases. Label and color scales of the score plot are reported

**Fig. 5:**
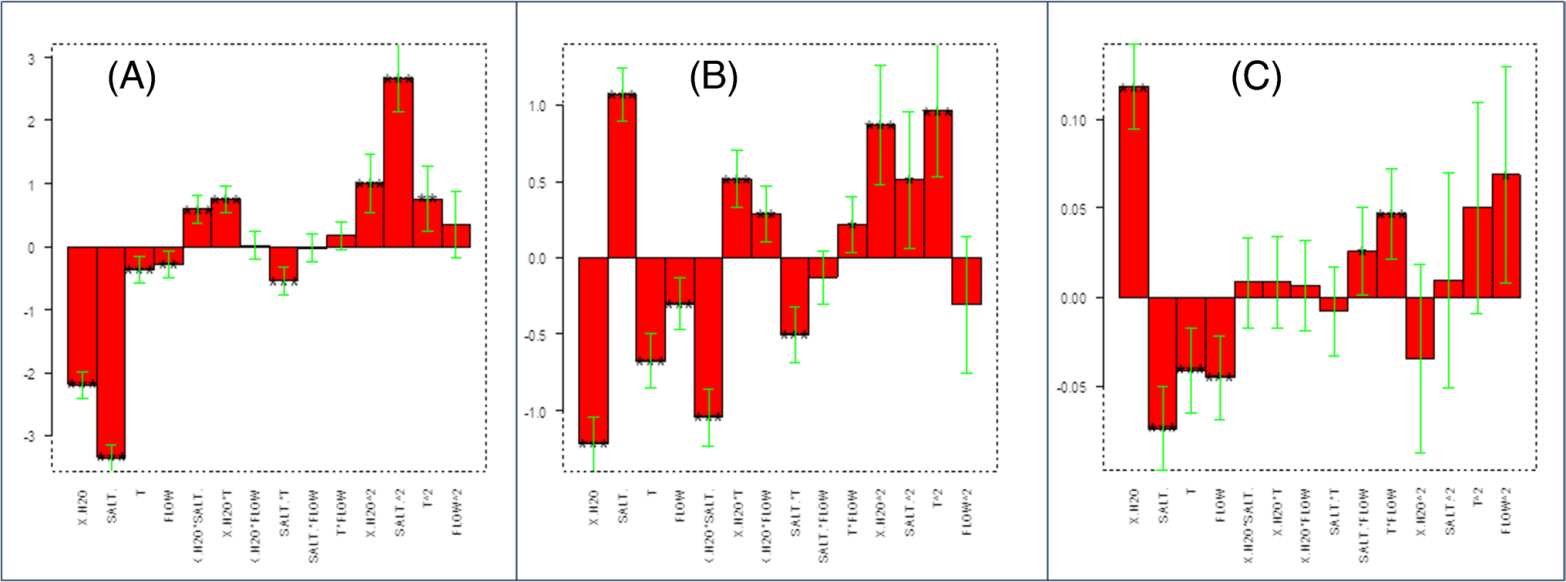
Examples of coefficient plots of chromatographic resolution. Models similar to ASA-CLBT (**A**), CAFF-SCL (**B**), and HCTZ-CAFF (**C**) are found next to the tail, next to the head, or far from the arrow, respectively

More interesting information can be achieved by looking at the PCA regarding compound resolutions: again, the most significant variable was salt concentration. In fact, most of the responses are distributed on a sort of arc of circumference following the golden arrow in Fig. 4A. Given the correlation of the responses and their grouping in the loading plot, only some of the single response models were computed, namely those considered representative of the identified “clusters.” At the tail of the above-mentioned golden arrow, models are similar to that reported in Fig. 5A (“group A” including most of the resolutions involving COCA or ACS), but moving along it, salt concentration coefficients increase gradually until models become Fig. 5B-like at its head (“group B,” mostly resolutions of SCL or ASA with other compounds). Moving away from this arc of circumference, there are the resolutions between dimethylxanthines and those of HCTZ with them (“group C”). What changes in these models is the coefficient of the A%, which gradually increases from the very negative values within the arrow to positive values far from it. Indeed, the score plot (Fig. 4B) shows that experiments located on the right side of the quadrant were mainly characterized by low A%. This meant that in these experiments, high values for the variables positioned along the “golden arrow” were observed, namely higher resolutions at lower A%. The farthest models from the arrow were characterized by coefficient plots like that shown in Fig. 5C, in which the coefficient for the A% was positive. A complete attribution to the defined groups can be found in Supplementary information (Table S.7).

This information was useful to understand the phenomenon and to optimize it; the goal, as in most chromatographic applications, was contextually maximizing resolutions and minimizing analysis time (represented by RT_SLBT_, the most retained analyte), by working on the most influencing variables, namely salt concentration and A%. Since different effects were observed, the optimal conditions were rather different depending on the “group” to which the response belongs. For example, increasing the percentage of eluent A would be ideal for the group C resolutions and RT_SLBT_, but most of the other resolutions were higher at the − 1 level of A%, implying a longer time of analysis. In order to keep satisfactory group C resolutions, the salt concentration was fixed at the − 1 level. This was also good for group A resolutions, but unideal for group B ones. Luckily, most resolution values belonging to group B were anyway satisfactory.

**Fig. 6:**
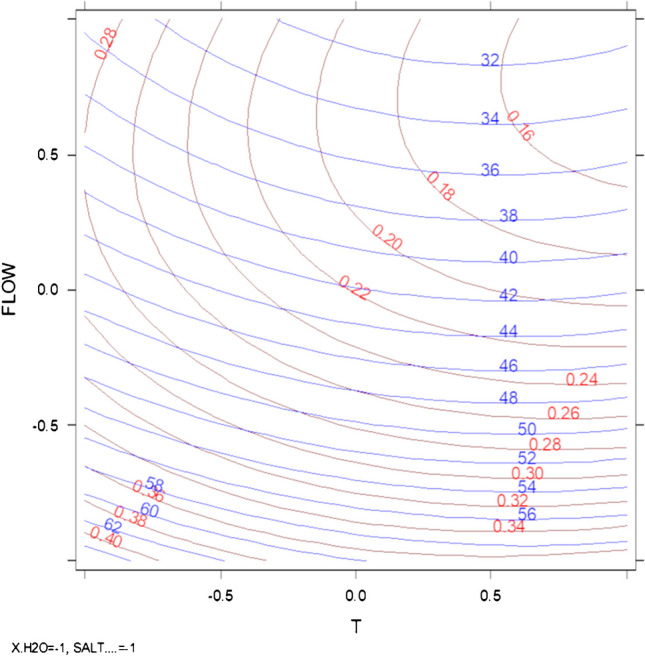
Overlapped contour plot of the effect of flow and temperature on SLBT's RT (in blue) and TFL-PRX resolution (in red) obtained by fixing the level of A% and that of salt concentration (both at − 1)

It must be noted that only two responses were particularly important but in contrast: RT_SLBT_ and R_TFL-PRX_, which presented different behaviors with respect to the investigated variables. The first is crucial because it represented the time of the analysis, being SLBT the last compound eluted in each tested condition; while *R*_TFL-PRX_ (whose coefficients plot of its model is reported in Figure S.3) was critical due to the impossibility of distinguishing PRX from TFL with the employed MS/MS detection [59], thus making necessary to separate them chromatographically. The factors temperature and flow presented divergent effects on these two responses: Fig. 6 reports the overlapped iso-response curves for RT_SLBT_ and R_TFL-PRX_, at fixed values of salt concentration and A%, which shows that achieving a higher resolution between PRX and TFL automatically implies a longer time of analysis.

#### Final considerations for the definitive chromatographic method

What seemed to be the better starting conditions for a complete chromatographic separation of the 12 target analytes (0% of eluent A with 0.2 mM of formate at a flow rate of 0.1 mL min^−1^ and a column temperature of 25 °C) required a rather long isocratic elution (RT_SLBT_ > 60 min). Thus, the development of a gradient elution was tested, also due to the necessity of a column “washing” during the analysis of real samples. In particular, a method involving both solvent and flow gradient was implemented, to try to speed up the analysis, without compromising the chromatographic resolution among TFL and PRX.

In the section “Plackett–Burman design—screening of the experimental variables,” it was clearly stated that acetate buffer was selected for the following DoE since it guaranteed a better sensitivity for negative-ionizing compounds. Nevertheless, when applying a combined flow and solvent gradient, an extensive suppression of the SLBT signal occurred (peak area more than 100-fold lower than that observed in isocratic conditions). This was partially expected and explained by the coefficients of water percentage and flow in determining SLBT peak area (Table S.6). Nonetheless, by applying the same initial conditions and gradient, but with formate buffer instead of acetate one, a higher sensitivity was immediately restored. Even if not evaluated through the DoEs, this could be ascribed to an interaction between these variables. Moreover, it has been previously demonstrated that autoclaving SLBT in acetate buffers causes its degradation [60].

Even though the negative-ionizing compounds showed better sensitivity with the acetate buffer, thus firstly selected for the D-optimal investigation, the observed suppression of the SLBT signal was not acceptable. Hence, formate buffer was used in the definitive method reported in “Instrumental analysis.”

### Development of sample treatment strategy—SALLE approach

The first sample treatment tested was SHS-HLLE with TEA as a tunable solvent. At low temperatures (4 °C) and at neutral/acidic pHs, TEA is miscible with water, obtaining the single-phase solution; through pH (by adding NaOH) and temperature changes, phase separation is induced [22]. The procedure was revealed to be quite easy and quick, but after a few trials, the low recoveries of the selected analytes (< 40%) and the incompatibility of TEA with MS systems [61] forced to change strategy.

The second approach chosen was SALLE, which exploits the phase separation of ACN from an aqueous phase occurring at the high ionic strength of the solution. A general procedure involves the preparation of a homogenized single-phase solution, which is added with salts, causing the phase separation. To guarantee a stratification of the organic layer, a centrifugation step is usually performed prior to withdrawing the surnatant, which is then filtered and properly diluted (or concentrated) before analysis. The 36 SALLE experiments related to the D-optimal DoE described in “Design of experiments” were performed on blank aqueous matrices spiked with the considered analytes. Each experiment was evaluated in terms of the recovery and matrix effect of each analyte, and these were used as model responses. Since more than 68% of the responses were in the 50–150% range, method accuracy was generally satisfactory for all the selected analytes, meaning that the changes applied with the DoE were often non-significant. In fact, the chemometric models built on these data were often poor, explaining low or even no variance (more information about these models is summarized in Table S.8). The computed models did not give any suggestion on how to further optimize the extraction process, but the overall results suggested that it is robust: when processing samples by routine analysis, small uncontrolled changes can occur, but they probably have a low impact on the experimental results [35, 50]. Even if it was not the aim of this DoE, ad hoc DoEs can be employed also to validate methods checking their robustness, by small but deliberate (and controlled) changes of some experimental variables [35, 50].

Still, five experimental conditions optimized the recovery and matrix effect values (the highest number of responses between the ideal range, 70–120% [50]). Among them, the one nearest to the experimental domain’s center was selected since it guarantees greater robustness [47].

### Method performances

As mentioned in the section “Evaluation of method performances,” both instrumental performances of the optimized separation method and the sample processing protocol are assessed and summarized in Table 3.

**Table 3:** Method performances

Analyte	*R*^2^	Linear range [μg L^−1^]	LOD [μg L^−1^]	LOQ [μg L^−1^]	Intra-day precision (lvl 1)^*a*^	Intra-day precision (lvl 2)^*b*^	Inter-day precision (lvl 1)^*a*^	Inter-day precision (lvl 2)^*b*^	R% (*n* = 6, ± std. dev.)	ME% (dil10k)	Procedural precision (*n* = 6, as RSD%)
ACS	0.9996	LOQ-50	0.02	0.07	9%	1%	27%	19%	51 ± 3%	108%	6%
FRSM	0.9996	LOQ-50	0.1	0.4	8%	8%	19%	16%	79 ± 9%	103%	11%
ASA	0.9996	LOQ-20	0.05	0.2	9%	3%	14%	9%	ND^c^	92%	ND^c^
HCTZ	0.9992	LOQ-20	0.01	0.04	8%	6%	19%	16%	84 ± 8%	110%	10%
CAFF	0.9998	LOQ-50	0.3	0.8	NC	6%	NC	6%	79 ± 9%	95%	11%
TFL	0.9978	LOQ-20	0.04	0.1	6%	6%	14%	12%	61 ± 4%	97%	6%
SCL	0.9989	LOQ-20	0.1	0.4	10%	2%	21%	10%	107 ± 10%	102%	9%
TBR	0.9998	LOQ-20	0.05	0.2	6%	5%	7%	7%	40 ± 1%	98%	2%
PRX	0.9982	LOQ-50	0.02	0.08	7%	5%	15%	9%	50 ± 1%	104%	1%
COCA	0.9999	LOQ-50	0.01	0.04	2%	1%	11%	12%	71 ± 6%	96%	8%
CLBT	0.9991	LOQ-20	0.02	0.05	3%	1%	28%	31%	88 ± 7%	93%	8%
SLBT	> 0.9999	LOQ-20	0.3	1	NC	1%	NC	40%	52 ± 4%	89%	8%

^a^lvl 1 concentration was 0.5 μg L^−1^ for every analyte but COCA (0.05 μg L^−1^). These precisions were not assessed for CAFF and SLBT since their LOQs were above the lvl 1 concentration employed

^b^lvl 2 concentration was 20 μg L^−1^ for every analyte except for COCA (2 μg L^−1^)

^c^Data not determined due to ASA’s degradation during the sample processing

In brief, the linear ranges cover at least two orders of magnitude except for CAFF, SCL, and SLBT, due to their higher LOQs. Anyway, linearity was always more than satisfactory within the considered ranges (*R*^2^ ≥ 0.9978). In-matrix LODs and LOQs were evaluated with two methods; values obtained by Eq. 2 (see Table S.9) were the most conservative (the higher ones) [50], ranging from 0.01 to 0.3 μg L^−1^ and from 0.04 to 1 μg L^−1^, respectively. They substantially corresponded to the instrumental values since, in the diluted matrix, ME was generally approximately 100%. Considering both the dilution factor due to the processing (from 120 mg of DS to 1.2 mL of ACN) and the one performed due to the high concentration within the samples (diluted 1- to 50-thousand times), the calculated LODs and LOQs in the original matrices are not reported, since lower concentrations can be easily determined, by diluting less. Still, in our case, extracts at lower dilutions (down to 100-fold dilution) were analyzed but a more significant matrix effect occurred (Table S.10) and no further analytes were detected.

Regarding specificity, the proposed method showed enough identification points for all compounds, based on the EU 2021 808 directive [62], which recommends 3–4 identification points to guarantee high confidence in identity confirmation of target analytes. Indeed, the RT and each MRM transition provided 1 and 1.5 identification points, respectively, thus giving 5 points for each compound except PRX, TBR, and TFL, for which a single MRM transition was monitored (and thus 3.5 points were achieved). Moreover, the ratios among the quantifier and qualifier MRM transitions were checked in all samples: values within ± 30% of the ones observed in neat standards were considered acceptable for confirmatory purposes [51].

Intra- and inter-day precision was assessed as RSD% of neat standards replicates; results are reported in Table 3. Intra-day precision was under 10% for the lower level and 8% for the higher one, while inter-day precision was generally lower, with RSD% up to 28% and 40% for the lower and higher level, respectively. Therefore, calibration standards were always included in the analysis batches.

The SALLE approach allowed a good recovery for the targeted analytes, in the range 50–110%, except for TBR. Considering the high polarity of these compounds, even the lower recoveries of some of them from the aqueous phase (TBR, ACS, PRX, and SLBT) were considered acceptable. Still, the quantitations of the analytes detected in real samples that presented a recovery significantly outside the ideal range of 70–120% were corrected for their own recovery [50], as reported in Table 3. Furthermore, as highlighted in Table 3, a good repeatability of the procedure was obtained, which allows reliable corrections for the recovery values. The accuracy and precision of the whole analytical method were not assessable for ASA, due to its well-known degradation [63]. Since a great dilution factor was employed for the analysis of real samples, ME is always acceptable and almost negligible, ranging from 89 to 111%.

This SALLE methodology was then compared with other studies concerning the extraction of compounds from DS (including at least one analyte in common). Regarding the amount of sample employed for each replicate, there are studies that require less [9], more [64], or even exactly the same quantity [14] of DS. All these works evaluated the content of a variable quantity of compounds in about a dozen of DS. Still, among the considered methods, our proposed SALLE procedure is the quickest and simplest one, involving less than a quarter of an hour to process a sample, doubling [14, 64] or increasing about tenfold [9] the productivity of the procedure and avoiding long times of ultrasonication extractions [14, 64], or laborious manual procedures like solid-phase extractions [9]. From a chromatographic point of view, some methods are quicker [9, 14] and others slower [64], also depending on the specific separation strategy and the specificity of the detector employed. In fact, it has already been proved that HILIC and RPLC can be considered as orthogonal separation strategies when dealing with doping-related studies [33]. Finally, the greenness of these four methodologies was estimated through the AGREE software, and the SALLE procedure developed in this work obtained a slightly higher score than the other studies: 0.74 [this work], 0.70 [9], 0.68 [14], 0.66 [64]. The detailed scorings are reported in Figure S.4 (A–D).

### Real samples

#### Target analytes

The processing of the eight real samples allowed the detection and quantification of some target compounds, detailed in Table 4. The only analyte with a declared amount on the DS labels was CAFF in C1 (61 mg g^−1^), C2 (220 mg g^−1^), C3 (not clearly), and P1 (350 mg g^−1^); the presence of the other methylxanthines was deduced from the plant extracts ingredients known to contain them, like *Camellia sinensis* (Green Tea), *Paullinia cupana* (Guaranà), and *Ilex paraguariensis* (Yerba-maté), while the artificial sweeteners were just labelled as “other ingredients.” Therefore, quantitative comparison with the labelled amounts can be done for CAFF only.

**Table 4:** Quantified target analytes within the samples; when not reported differently, 10-thousand times dilution was considered. ND stands for “not detected” which means < LOD even at the lower dilution (100-fold)

DS	ACS^a^	CAFF	TFL^a^	SCL	TBR^a^	PRX^a^
μg g^−1^						
C1	ND	(41.0 ± 0.6) × 10^3 b^	70 ± 2	ND	111 ± 2	27.6 ± 0.4
C2	ND	(192.2 ± 0.5) × 10^3 b^	20 ± 3^c^	ND	(0.58 ± 0.03) × 10^3^	37 ± 3
C3	ND	(106 ± 3) × 10^3 b^	18 ± 2^c^	ND	45 ± 4	52 ± 1
C4	ND	ND	ND	ND	ND	ND
C5	ND	ND	ND	ND	ND	ND
P1	(7.66 ± 0.04) × 10^3^	(27 ± 1) × 10^3 b^	ND	(8.5 ± 0.3) × 10^3 b^	ND	19.6 ± 0.2
P2	620 ± 4	126 ± 4	ND	(1.95 ± 0.06) × 10^3^	(2.04 ± 0.01) × 10^3^	ND
P3	ND	99 ± 5	ND	(0.5 ± 0.1) × 10^3^	(1.71 ± 0.07) × 10^3^	ND

^a^Experimental results corrected for the analyte recoveries when lower than 70%, as reported in Table 3

^b^Quantification using a 50-thousand times dilution

^c^Quantification using a 1-thousand times dilution

Quantitation results of all the samples were not directly compared due to the differences in the dosage, serving mode, and usage indications. C1, C2, and C3 were caffeine-based pre-workout DS, also containing several plant extracts like guarana and green tea extracts, explaining and justifying the presence of each of the 4 methylxanthines [65]. Furthermore, the declared dose of caffeine within C1 and C2 was clearly labelled and reasonably agreed with the quantified amounts. By contrast, C3 presented a less clear label, which resulted in a difficult comparison with the experimental data. Nevertheless, considering the number of capsules suggested on the labels (4 caps for C1, 1 cap for C2, and 1–2 caps for C3), their caffeine dosages were very close but within the recommended maximum single-dose intake of 200 mg [43], which is also the maximum licit dose for DS sold in Italy [66]. Both C4 (plant extract–based) and C5 (protein-based) were DS in a capsulated form that did not report any of the target analytes in their labels, and none of them was indeed detected.

Since P1, P2, and P3 were in a powdered form which requires a water dissolution before drinking, they need to taste good. Each of them declared on their label at least one sweetener between ACS and SCL, which was correctly detected in the extracts. As an example, a chromatogram showing the MRM transitions of ACS present in P2’s diluted extract is reported in Figure S.5. Still, the amounts of these compounds were not specified on the labels and thus a comparison was not possible, but the detected amounts are consistent with that required to obtain a sweet taste (considering their sweetening power [67] and the suggested powders dilutions). P1 was again a pre-workout caffeine-based DS, confirmed by the quantitation of this methylxanthine, while P2 and P3 were protein-based DS containing powder cocoa to enhance taste; this explained why both TBR and CAFF were detected in the extracts. Moreover, the experimental TBR/CAFF ratios (in the range of 16.2–17.3) in these DS are reasonable with the natural ratios found in cocoa powders in the literature (5.8–19.8) [68], suggesting that none of them were artificially added as pure compounds in the DS’s formulations. For this kind of product, the corresponding single-dose intake of caffeine depends on the amount of DS consumed following the instructions reported on its label: since P1’s label requires dissolving 9 g of DS for a serving, this would exceed the Italian legislative limits of 200 mg of CAFF [66], as already deducible by the label itself. On the contrary, P2 and P3 required 32 and 33 g of DS per serving, respectively, but the quite low amount of caffeine present in these DS is absolutely within the limits.

Similar results were obtained by Helle et al. [6] in Norway, who found 4 DS out of the 93 analyzed containing an illicit content of caffeine for the single-dose serving (with respect to the 300 mg of the Norwegian limit). Unfortunately, they did not report details on the processing of the samples and thus it was not possible to compare that aspect.

#### Tentative identification of clenbuterol analogues

Even though none of the considered illicit drugs was detected in the DS, one of them presented a commercial name strongly evoking clenbuterol (even if it was not detected in the extract). This compound is the most common of a class of anabolic, β_2_-agonists, which only differ in the nitrogen substituents “**R**” [17], which is, for CLBT, the tert-butyl group. Therefore, a suspect screening approach was implemented. The typical behavior of this group of compounds in ESI is the formation of the pseudo-molecular ions [M + H]^+^, which undergo fragmentation by losing at first a water molecule and then the nitrogen substituent as it is or, less commonly, as **R = NH** (neutral loss of unsaturated molecules) giving the product ions at *m/z* = 203.0 and 188.0 [69]. This fragmentation can keep going on, producing other product ions (like those at *m/z* = 168.0 and 132.1) independently from their precursor [17, 69]. Thus, several product ions are theoretically common to all these analogues, making the performance of a PcIS a viable option for screening. This evaluation was performed by eluting the DS extracts with the same gradient set for the target analytes and by monitoring if any precursor ion was able to produce the most intense product ions of CLBT.

An interesting peak was observed at RT = 19.9 min, due to the precursor ions at *m/z* = 262.1 and 263.1, producing both the ions at *m/z* = 203.0 and 132.1. Thus, an MRM and a PdIS were performed, to achieve further information. The MRM analysis confirmed the fragmentation of the two precursor ions observed, producing both the two product ions, while PdIS revealed a further fragmentation to *m/z* = 103.0 and 115.0. To the authors’ knowledge, the production of these ions for this kind of compound has never been reported before [17, 70], but it can be rationalized respectively as further loss of CH_2_ = NH (MW = 29 Da) and ammonia (MW = 17 Da) from the ion at *m/z* = 132.1.

**Fig. 7:**
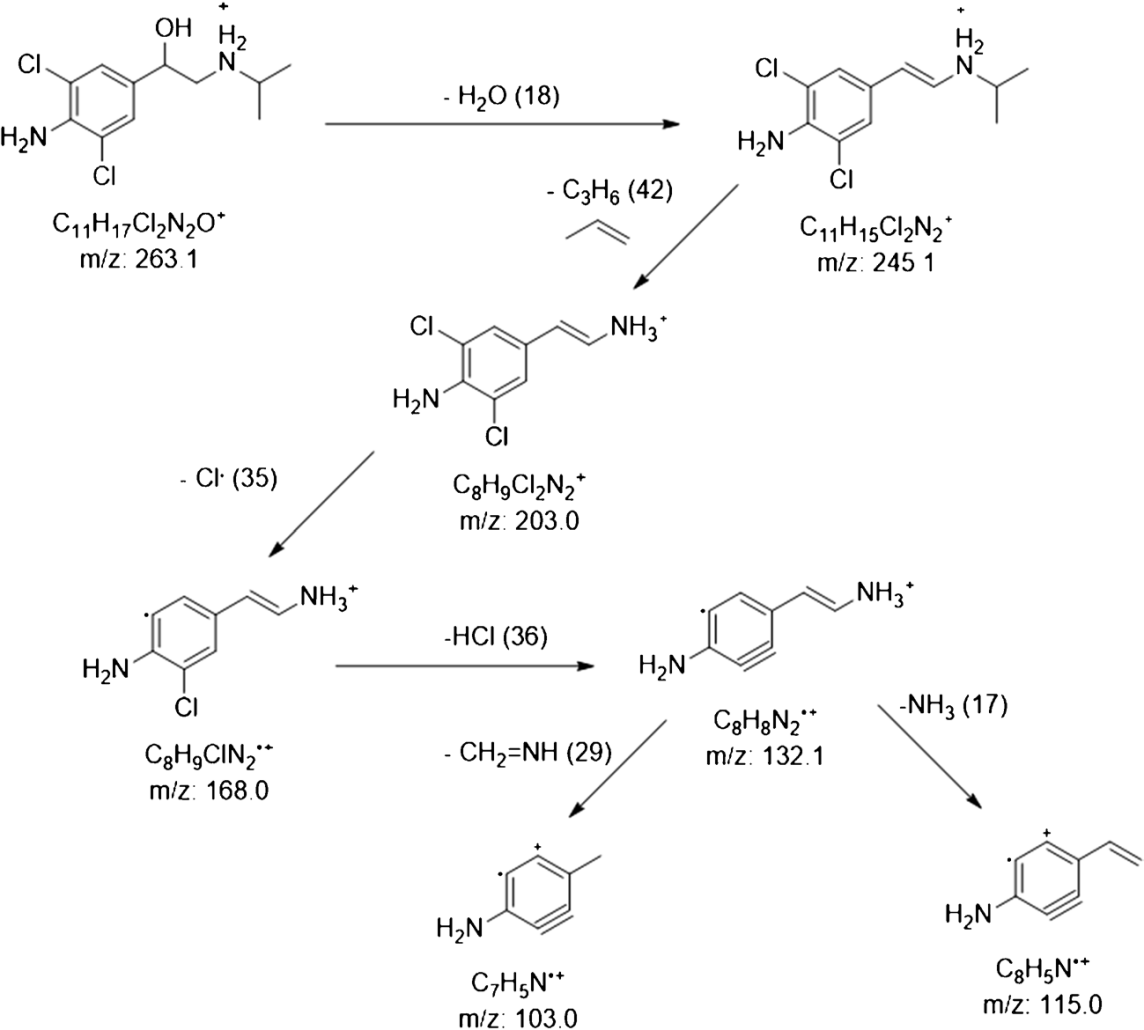
Suggested ESI + /QQQ fragmentation pathway of clenproperol, one of the possible structures hypothesized to be responsible for the analytical signal detected in C1 extract

Since a single chromatographic peak was observed by extracting the current of the ions at *m/z* = 262.1 and 263.1, the former may be interpreted either as an unusual production of the molecular ion in ESI [M]^•+^ of the same compound ionizing through protonation ([M + H]^+^) [71, 72] or as an in-source loss of H•, thus indicating a single eluting species. Hence, these data can be rationalized as the presence of a possible CLBT analogue with a -C_3_H_7_ nitrogen substituent, whose possible fragmentation pathway is shown in Fig. 7. An additional fact supporting the hypothesis was the observed elution order in the HILIC analysis (more polar compounds are more retained on the column than less polar ones). The hypothesized propyl group as nitrogen substituent instead of the tert-butyl should imply a slightly higher polarity of the molecule, which fits the elution order observed (RT_CLBT_ = 19.0 min, RT_analogue_ = 19.9 min).

All this information allowed obtaining of enough identification points to assign the detected signal between the L2 (probable structure by diagnostic evidence) and L3 (tentative candidates identified as a substructure and/or class) levels according to Röhler et al. [73], also because of the impossibility to distinguish among some possible isomers (for example, a n-propyl and an iso-propyl group would have led to the same fragmentations). Further analyses could increase the confidence points, but a reference standard is still necessary to confirm the identity of the unknown detected compound.

## Conclusions

The first step of this work deeply exploited the DoE to fully understand the retention mechanism of the targeted analytes on the selected diolic HILIC column. Since the experimental variables presented divergent effects on the responses, it was necessary to find the best compromise. The critical separation of TFL and PRX forced some of the starting conditions of the chromatographic separation. Furthermore, when trying to accelerate the analysis by performing a gradient, the strong suppression in SLBT sensitivity required to change buffer, restoring the formate one, which was excluded from the D-optimal DoE investigations. Once the instrumental method was fully developed, the sample treatment strategy was studied as well via a chemometric approach. The optimized SALLE allowed to satisfactorily extract the polar analytes from the DS matrix and thus it was used to process the real samples. The high dilution factor applied, due to the high concentration expected, implied almost no matrix effect, quantifying six legal ingredients within several DS. Summarizing the obtained results, none of the targeted illicit compounds were detected (also at lower dilutions) and none of the products was clearly mislabelled, while several legal compounds were correctly detected and quantified. Still, one of the caffeine-based products revealed a single-dose CAFF content higher than that allowed by Italian authorities [66]. In conclusion, the developed strategy can be successfully used to quantify both some polar legal ingredients and illicit dopant compounds with good performances and reliability. This can be useful for several aims, such as checking the correct labelling of the DS and preventing or confirming possible unintended doping due to DS contamination or faking. Furthermore, a sequence of PcIS, PdIS, and MRM scans was positively applied detecting and tentatively identifying a structural analogue of CLBT, for which further investigations will be planned.

### Supplementary Information

Below is the link to the electronic supplementary material.

Supplementary file1 (XLSX 1423 KB)
